# Effect of Chronic Administration of 5-(3-chlorophenyl)-4-Hexyl-2,4 -Dihydro-3*H*-1,2,4-Triazole-3-Thione (TP-315)—A New Anticonvulsant Drug Candidate—On Living Organisms

**DOI:** 10.3390/ijms22073358

**Published:** 2021-03-25

**Authors:** Anna Makuch-Kocka, Marta Andres-Mach, Mirosław Zagaja, Anna Śmiech, Magdalena Pizoń, Jolanta Flieger, Judyta Cielecka-Piontek, Tomasz Plech

**Affiliations:** 1 Department of Pharmacology, Faculty of Health Sciences, Medical University of Lublin, 20-093 Lublin, Poland; tomasz.plech@umlub.pl; 2 Isobolographic Analysis Laboratory, Institute of Rural Health, 20-090 Lublin, Poland; andres.marta@imw.lublin.pl (M.A.-M.); zagaja.miroslaw@imw.lublin.pl (M.Z.); 3 Sub-Department of Pathomorphology and Forensic Veterinary Medicine, Department and Clinic of Animal Internal Diseases, University of Life Sciences in Lublin, 20-612 Lublin, Poland; anna.smiech@up.lublin.pl; 4 Department of Analytical Chemistry, Faculty of Pharmacy, Medical University of Lublin, 20-093 Lublin, Poland; magdalena.pizon@umlub.pl (M.P.); jolanta.flieger@umlub.pl (J.F.); 5 Department of Pharmacognosy, Faculty of Pharmacy, Poznan University of Medical Sciences, 61-781 Poznań, Poland; jpiontek@ump.edu.pl

**Keywords:** epilepsy, hepatotoxicity, nephrotoxicity, 1,2,4-triazole-3-thione derivatives, CYP450 enzymes, antiepileptic drugs

## Abstract

About 70 million people suffer from epilepsy—a chronic neurodegenerative disease. In most cases, the cause of the disease is unknown, but epilepsy can also develop as the result of a stroke, trauma to the brain, or the use of psychotropic substances. The treatment of epilepsy is mainly based on the administration of anticonvulsants, which the patient must most often use throughout their life. Despite significant progress in research on antiepileptic drugs, about 30% of patients still have drug-resistant epilepsy, which is insensitive to pharmacotherapy used so far. In our recent studies, we have shown that 4-alkyl-5-aryl-1,2,4-triazole-3-thiones act on the voltage-gated sodium channels and exhibit anticonvulsant activity in an MES (maximal electroshock-induced seizure) and 6Hz test in mice. Previous studies have shown their beneficial toxic and pharmacological profile, but their effect on a living organism during chronic use is still unknown. In the presented study, on the basis of the previously conducted tests and the PAMPA (parallel artificial membrane permeability assay) BBB (blood–brain barrier) test, we selected one 1,2,4-triazole-3-thione derivative—TP-315—for further studies aimed at assessing the impact of its chronic use on a living organism. After long-term administration of TP-315 to Albino Swiss mice, its effect on the functional parameters of internal organs was assessed by performing biochemical, morphological, and histopathological examinations. It was also determined whether the tested compound inhibits selected isoforms of the CYP450 enzyme system. On the basis of the conducted tests, it was found that TP-315 does not show nephrotoxic nor hepatotoxic effects and does not cause changes in hematological parameters. In vitro tests showed that TP-315 did not inhibit CYP2B6, CYP2D6, CYP3A4, or CYP3A5 enzymes at the concentration found in the serum of mice subjected to long-term exposure to this compound.

## 1. Introduction

Epilepsy is one of the most common neurological diseases in the world. It is estimated that approximately 65 million people in the world, or approximately 1% of the population, suffer from epilepsy. Currently, the number of people suffering from the active form of epilepsy is around 5–10 people in 1000 [1,2,3]. Epileptics are at an increased risk of death (about 1.6–4.1 times higher compared to the general population), which is associated with epileptic seizures, epileptic state, suicide, or sudden unexpected death in epilepsy (SUDEP) [4].

Treating epilepsy is primarily based on properly selected pharmacotherapy. Currently used drugs do not have the ability to inhibit epileptogenesis, they only show a symptomatic effect. The first-line treatment of epilepsy is the use of so-called classic antiepileptic drugs (AEDs). According to statistics, they are effective, giving full control of seizures, in about 60% patients with epilepsy. Additionally, polytherapy turned out to be effective in the next 15–20% of cases. Unfortunately, nearly 30% of patients suffer from drug-resistant epilepsy (DRE) [5]. New drugs available on the pharmaceutical market, such as gabapentin, pregabalin, rufinamide, lamotrigine, vigabatrin, topiramate, or felbamate are characterized by better pharmacokinetics and fewer side effects compared to classical antiepileptic drugs, and slightly increased effectiveness in DRE [6]. A serious problem associated with drug resistance is the higher mortality rate in patients with drug-resistant epilepsy in comparison with other patients with epilepsy. Recurrent epileptic seizures increase secondary epileptogenesis, which increases the frequency of seizures. Frequent generalized seizures have numerous medical and social consequences, e.g., increased risk of injuries and fractures, progressive memory disorders, progressive cognitive impairment, and increased risk of mental disorders. The social consequences of drug-resistant epilepsy include social stigmatization, job loss, the costs of treatment of the co-morbidities and complications of epilepsy, and the costs of long-term institutional care [7].

In our recent studies, we have demonstrated that 1,2,4-triazole-3-thione derivatives represent a group of promising antiepileptic drug candidates [8,9,10,11]. Such compounds were active against tonic-clonic seizures and in an animal model of drug-resistant epilepsy [12]. Moreover, we have also found that the substitution of alkyl moiety with an aryl group resulted in compounds endowed with both potent anticonvulsant effect [13] and beneficial interactions with classical antiepileptic drugs [14,15]. Other authors also proved that compounds based on a 1,2,4-triazole scaffold possess anticonvulsant activity in a broad spectrum of animal models of epilepsy [16,17]. It turned out that the anticonvulsant activity determined in animal models of epilepsy is closely correlated to the interaction of 1,2,4-triazole-3-thione derivatives with voltage-dependent sodium channels [8,18]. Our earlier results also showed that the investigated class of compounds was devoid of genotoxic effects when tested in HepG2 cells [18]. However, it should be emphasized that all these previously performed studies concerned 1,2,4-triazole derivatives focused only on the preliminary screening of their anticonvulsant properties. In those studies, the effect of long-term use of the compounds on living organisms has not been considered thus far.

In the presented study, on the basis of the previously conducted experiments and the PAMPA BBB test, we selected one 1,2,4-triazole-3-thione derivative—TP-315—for further studies aimed at assessing the impact of chronic use of the test compound on a living organism. After long-term administration of TP-315 to Albino Swiss mice, the effect of the compound on the functional parameters of internal organs was assessed by performing biochemical, morphological, and histopathological examinations. The possible interaction of TP-315 with selected isoforms of the CYP450 enzyme system was also determined.

## 2. Results and Discussion

### 2.1. Selection of 1,2,4-Triazole-3-Thione Derivative as a Potential Antiepileptic Drug to Determine the Effects of Chronic Administration to a Living Organism

**Figure 1:**
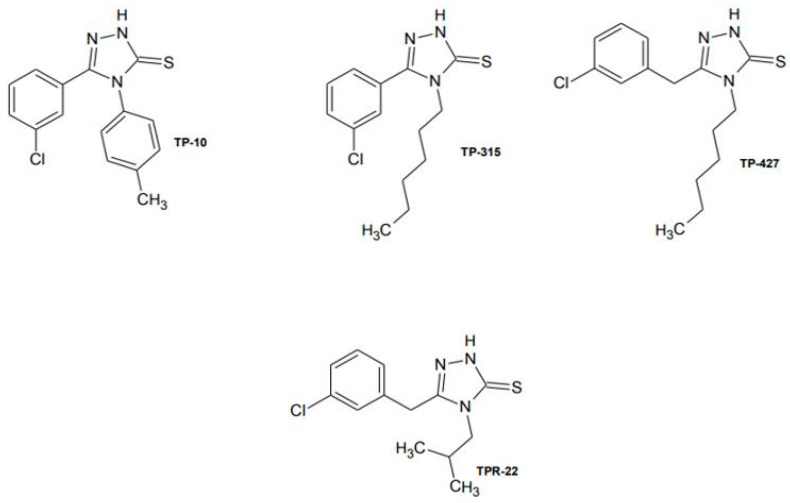
Chemical structures of 1,2,4-triazole-3-thione derivatives selected for further investigations. TP-10 5-(3-Chlorophenyl)-4-(4-methylphenyl)-2,4-dihydro-3*H*-1,2,4-triazole-3-thione, TP-315 5-(3-Chlorophenyl)-4-hexyl-2,4-dihydro-3*H*-1,2,4-triazole-3-thione, TP-427 5-(3-Chlorobenzyl)-4-hexyl-2,4-dihydro-3*H*-1,2,4-triazole-3-thione, and TPR-22 5-(3-Chlorobenzyl)-4-isobutyl-2,4-dihydro-3*H*-1,2,4-triazole-3-thione.

Out of all 1,2,4-triazole-3-thione derivatives with anticonvulsant activity we have synthesized so far, the four most promising compounds have been selected that may be potential antiepileptic drugs: TP-10, TP-315, TP-427, and TPR-22 (Figure 1).

In order to select one 1,2,4-triazole-3-thione derivative for studies determining the effects of chronic administration of the selected compound on a living organism, the results of previously published studies were compared, and the PAMPA BBB test was performed. The anticonvulsant activity of the tested compounds (obtained from the MES (maximal electroshock-induced seizure) and 6 Hz tests), their neurotoxicity (obtained from chimney and rotarod tests), and their affinity towards batrachotoxin-binding site on voltage-gated sodium channels were analyzed (Table 1).

The key criterion for selecting a compound for further studies was also the blood–brain barrier (BBB) permeability. The BBB is one of the main obstacles limiting AEDs from reaching the epileptic site in the brain. Very often, it causes a reduction in the effectiveness of the treatment or the need to increase the doses of AEDs. Increasing the doses of drugs may cause the occurrence of toxic side effects, which may pose a threat to the patient’s health [19,20]. In the case of compounds that are candidates for antiepileptic drugs, an important issue is their good permeability through the BBB.

The blood–brain barrier permeability studies results confirmed that four examined 1,2,4-triazole-3-thione derivatives can be classified as BBB+, since the compounds with Pe > 5.19 cm/s are characterized by good permeation through BBB [21]. The compounds also have much higher permeability across the BBB in comparison to valproic acid (a standard drug used in epilepsy treatment). TP-315 has the highest permeability across the BBB in comparison to TP-10, TP-427, and TPR-22 (Table 1). Moreover, TP-315 showed good anticonvulsant effects in the maximal electroshock-induced seizure (MES) model of generalized tonic-clonic seizures in humans and in the 6 Hz psychomotor seizure model of drug-resistant epilepsy (Table 2).

By analyzing the results of previously conducted studies and the results of the PAMPA BBB test, TP-315 was qualified for further research on the effects of chronic administration.

### 2.2. Effects of Chronic Administration of TP-315 on Living Organisms

Pharmacological treatment of epilepsy is difficult due to the numerous toxic effects of antiepileptic drugs used in standard therapy. The dose of AEDs for each patient must be determined individually to minimize the occurrence of side effects while reducing the risk of seizures. Very often, the concentration of the drug in the serum is monitored in patients and the hepatic (ALT, AST) or renal (urea, creatinine) parameters are determined and the blood count is performed. The purpose of these biochemical and morphological determinations is to check the function of the internal organs to prevent the toxic effects of antiepileptic drugs [23,24]. Carbamazepine and valproic acid are commonly used to treat epilepsy. Common side effects with valproic acid are elevated levels of aspartate aminotransferase (AST) and alanine aminotransferase (ALT), leukopenia, or thrombocytopenia. According to data from the World Health Organization (WHO), valproic acid is one of the three drugs that most often cause severe liver damage in patients and the need for a transplant [25]. Elevation of liver enzymes has also been reported during carbamazepine treatment [26,27,28,29]. Many antiepileptic drugs are sodium channel blockers, which, in addition to the brain tissue, can also travel to the kidneys, liver, lungs, or bone marrow, causing toxic effects [30,31]. An example of such a drug is phenytoin, the clinical use of which is severely limited as a result of chronic toxicity such as hepatotoxicity, leukopenia, or megaloblastic anemia [32,33].

When conducting research on newly synthesized compounds, which are potential candidates for antiepileptic drugs, the key, apart from their anticonvulsant activity, is to exclude their toxic effects on the patient’s body. Pharmacological treatment of epilepsy usually covers the whole life of the patient, so it is important that the administered drug does not cause severe side effects or toxic effects on internal organs such as the kidneys, liver, or bone marrow.

#### 2.2.1. Histopathological Examination

In order to check whether TP-315 induces nephrotoxic or hepatotoxic effects after long-term treatment, a histopathological examination of the kidneys and liver were performed.

**Figure 2:**
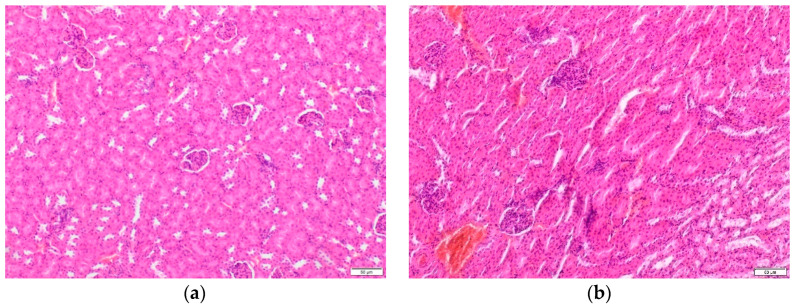
The histopathological structures of mouse kidney tissues after TP-315 treatment (hematoxylin and eosin staining(H&E) × 100). Photomicrograph of the first convoluted proximal tubules lined by a single-layered cuboidal epithelium (**a**). Photomicrograph of the second convoluted distal tubules (**b**).

There were no differences in the microscopic structure of kidneys from the control and experimental groups. The kidneys had normal cortical and medullary parenchyma. The first convoluted proximal tubules lined by a single-layered cuboidal epithelium were arranged regularly. A distinct nucleus surrounded by eosinophilic cytoplasm was visible centrally in the epithelial cells. The stellate lumen of the tubules was obscured by the brush border (Figure 2a). The second convoluted distal tubules were characterized by a regular round or oval lumen. The boundaries of the epithelial cells were weakly visible (Figure 2b).

**Figure 3:**
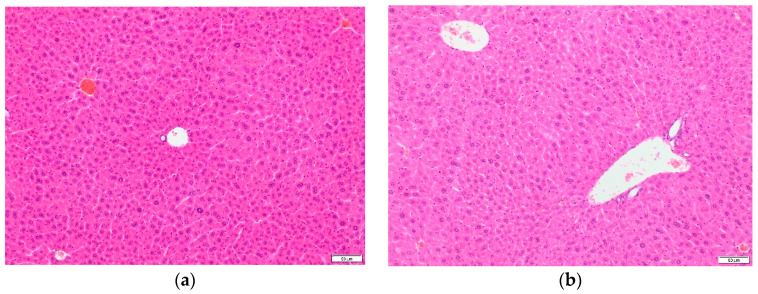
(**a**,**b**) The histopathological structures of mouse liver tissues after TP-315 treatment (H&E staining, × 100). Hepatocytes forming hepatic trabeculae arranged radially towards the central veins (**a**). The adjacent portobiliary spaces (**b**).

The microscopic picture of the liver as a normal organ without pathological changes was similar in the experimental and control groups. Hepatocytes with eosinophilic cytoplasm formed hepatic trabeculae arranged radially towards the central veins. (Figure 3a) The borders of the hepatic lobules were marked by lines connecting the adjacent portobiliary spaces. (Figure 3b). Cross-sections through arteries, veins, and interlobular bile ducts were visible in the spaces.

#### 2.2.2. Determination of Biochemical Parameters of Liver and Renal Function

**Figure 4:**
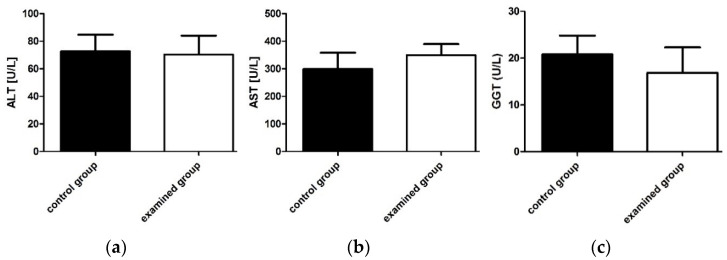
The differences in ALT (**a**), AST (**b**), and GGT (**c**) concentration in the serum of the mice. The data were plotted as the mean value ± standard error (SD). Statistical analysis was performed using an unpaired t-test (significance was accepted at *p* < 0.05). The differences in the values of the discussed parameters in the mice from the experimental group in comparison to the mice from the control group were not statistically significant.

Alanine aminotransferase (ALT), aspartate aminotransferase (AST), and γ-glutamyltranspeptidase (GGT) value alteration is an important indicator of the degree of liver damage. As can be seen in Figure 4, the mean values of the discussed parameters determined in the serum of test mice did not differ statistically significant from those determined in mice from the control group (*p* > 0.05). Based on the ALT, AST, and GGT values, it can be concluded that TP-315 does not show significant changes in the activity of liver enzymes, which suggests that it has no toxic effect on the liver.

**Figure 5:**
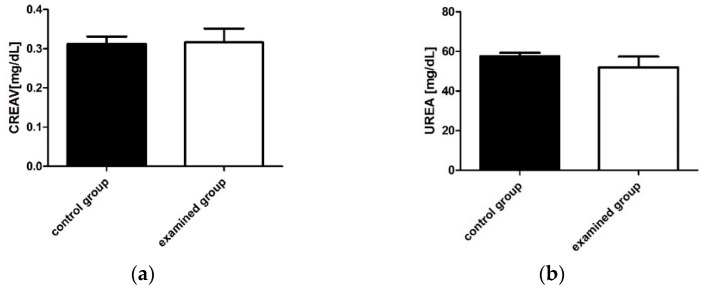
The differences in creatinine (**a**) and urea (**b**) concentration in the serum of the mice. The data were plotted as the mean value ± standard error (SD). Statistical analysis was performed using an unpaired t-test (significance was accepted at *p* < 0.05). The differences in the values of the discussed parameters in the mice from the experimental group in comparison to the mice from the control group were not statistically significant.

The effect of chronic administration of TP-315 on mice from the experimental group on the indices of renal function is presented in Figure 5. The differences in the mean concentration of urea and creatinine in the serum of mice administered the test compound compared to the values of these parameters in the serum of mice from the control group were not statistically significant (*p* > 0.05). Based on the values of the parameters described above, it can be concluded that TP-315 does not induce functional changes in the kidneys.

#### 2.2.3. Determination of Morphological Blood Parameters

In order to exclude the influence of chronic administration of TP-315 on the alteration of hematological parameters and potential myelotoxic effects, the blood parameters presented in Table 3 were assessed in mice from the experimental and control groups. The mean values of the determined parameters in the blood of mice administered TP-315 did not differ statistically significantly from the mean value of these parameters determined in the control group (*p* > 0.05). Based on the presented results, it can be concluded that chronic administration of TP-315 does not cause disturbances in the white blood cell and red cell systems.

Results are shown as mean ± SD (*p* < 0.05 was assumed statistically significant, unpaired t-test). The differences in the values of the discussed parameters in the mice from the experimental group in comparison to the mice from the control group were not statistically significant. Shortcuts used in Table 3: RBC, red blood cells; HGB, hemoglobin concentration; MCV, mean corpuscular volume; MCH, mean corpuscular hemoglobin; MCHC; mean corpuscular hemoglobin concentration; RDW-CV, red blood cell distribution width; RDW-SD, red blood cell distribution width standard deviation; HCT, hematocrit; PLT, platelet; MPV, mean platelet volume; PDW, platelet distribution width; PCT, plateletcrit; WBC, white blood cells; BAS, basophils; NEU, neutrophils; LYM, lymphocytes; MON, monocytes; EOS, eosinophile.

### 2.3. Effect of TP-315 on the Inhibition of Selected CYP450 Isoforms

In the development of new drugs, it is very important to establish the risk of drug interactions. With current antiepileptic drugs, drug interactions mainly involve pharmacokinetic processes resulting in the induction or inhibition of microsomal enzyme activity. The greatest risk of drug interactions is with the use of old generation antiepileptic drugs such as phenobarbital, phenytoin, or carbamazepine. In patients with epilepsy, the occurrence of drug interactions is particularly important because AEDs are used for a long time, usually throughout the patient’s life. The metabolic enzyme inducers are primidone, phenytoin, and carbamazepine—they influence the activity of the cytochrome CYP450 isoenzymes such as CYP2C19, CYP2C9, CYP3A4, and CYP1A2. New generation antiepileptic drugs have not so strongly expressed properties of inducing the metabolism of others drugs as old generation drugs, while oxcarbazepine is a weak inhibitor of CYP2C19 [34,35,36,37,38,39].

To exclude a significant effect of TP-315 on the induction or inhibition of individual CYP450 isoforms, experiments were conducted to determine the concentration of the test compound in the serum of mice that were chronically administered TP-315, and then enzyme tests were performed to check whether TP-315 at the determined concentration affected the activity of the CYP2B6, CYP2C19, CYP2D6, CYP3A4, and CYP3A5 enzymes.

#### 2.3.1. Quantification of TP-315 by HPLC-FL Method

The standard addition method (SAM) is considered to be one of the most straightforward methods for the elimination of the influence of interferences present in a complex matrix [40]. The SAM calibration curves of the recovered standard enable the determination of the intersection of a straight line with the *x*-axis, which is the value used for calculation of the targeted analyte concentration. Additionally, the influence of interferences on the results is eliminated by applying chromatography as a separation method [41,42,43,44].

**Figure 6:**
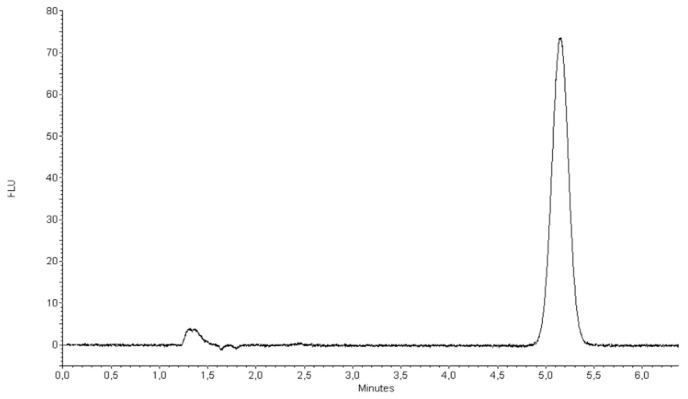
HPLC chromatograms obtained for TP-315 at the concentration of 0.06 ppm. The analysis was performed using a Zorbax Eclipse XDB-C18 column. The mobile phase was methanol (80%, *v/v*) and perchloric acid (0,1%, *v/v*). The flow rate of the mobile phase was 1 mL/min. The fluorescence detection was performed at excitation at 265 nm and emission at 411 nm. The injection volume was 20 μL.

During the previous experiments [45,46], the HPLC separation conditions were established, in order to obtain the optimized retention, system efficiency, and the detection limits. Currently elaborated conditions were modified by changing the mobile phase composition in the aim to adjust the chromatographic system to the new biological matrix. The best conditions were obtained using a C18 column with the mobile phase containing 80% methanol with the of addition 0.1% perchloric acid. Detection was monitored by a fluorescence (FL) detector for the identification and quantification of the analytes. Figure 6 presents a typical chromatogram obtained for this pattern. The fluorescence detection ensured the most advantageous limits of detection and quantification.

The retention time for TP-315 was 5.158 min. The applied system ensured sufficient efficiency expressed in the theoretical plates number (USP) (N = 25180/m) and peak symmetry (As = 0.9655). The limits of the detection under fluorescence detection were found to be 0.025 ng/mL, taking into account the 3:1 signal to noise ratio.

- Recovery examination

**Figure 7:**
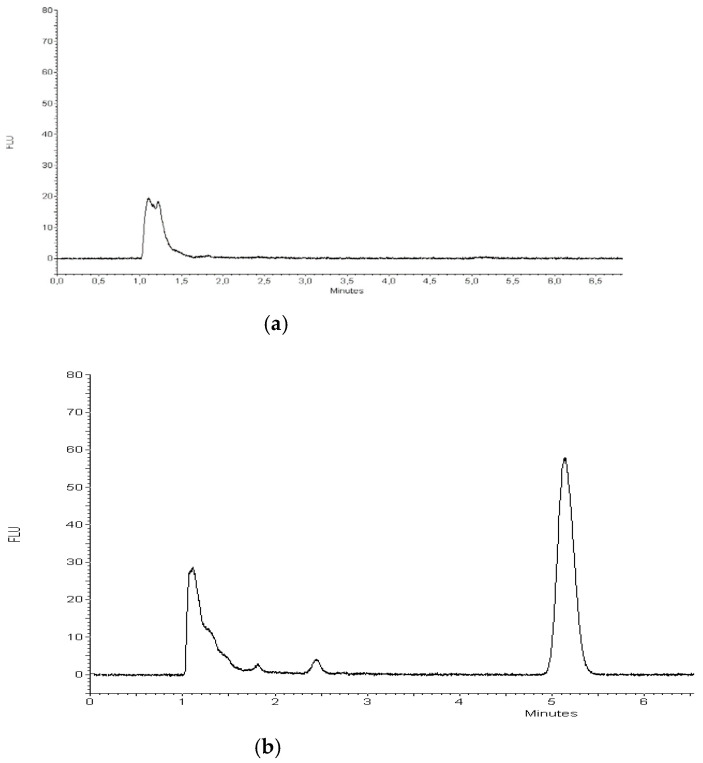
Representative chromatograms of blank serum sample (**a**), and serum sample spiked with TP-315 (0.18 ppm) (**b**), which were subjected to the SPE procedure. Conditions: stationary phase, Zorbax Extend-C18 (150 mm × 4.6 mm I.D; 5-_m); mobile phase, 80% MeOH/0.1% HClO4/water; flow rate, 1 mL/min; detection, ex = 265 nm, em = 411 nm.

The accuracy of the method was established by the standard addition method. The blank samples were spiked with the analyte standard solution at different concentration levels. These samples yielded a calibration curve with excellent linearity in the corresponding range with square correlation coefficient r2 >0.99. Figure 7 presents a representative chromatogram of blank serum sample (Figure 7a) and serum spiked with TP-315 standard at a concentration of 0.18 ppm (Figure 7b). As can be seen, no endogenous serum sample components were eluted at the retention time of TP-315 under the studied conditions. The mean recovery values determined for the QCs by SPE-HPLC-FL were in the range from 96.66% to 99.39% at certain concentration levels, and the obtained precision results were satisfactory. RSD% for repeatability was always lower than 5%. The obtained results are summarized in Table 4.

- Analysis of serum samples

The amount of the analyte in the sample was calculated from the individual calibration curve, prepared by linear regression. The mean measured concentration of TP-315 in the plasma of the mice was 14.52 ± 12.54 ng/mL (mean ± SD).

#### 2.3.2. Concentration-Dependent Screening of TP-315 on Enzyme Activity

**Figure 8:**
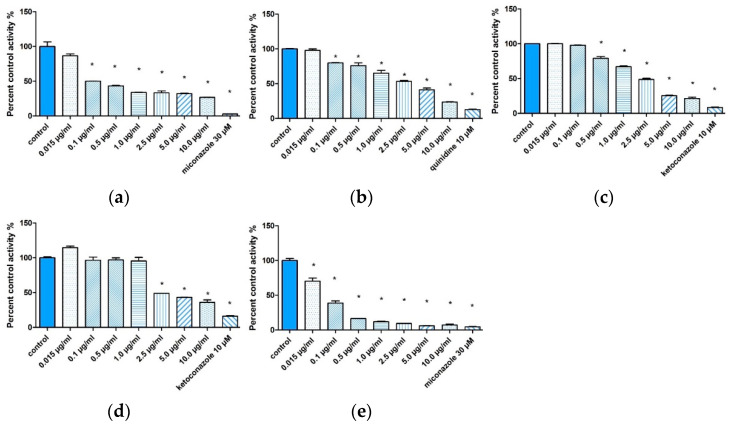
Screening of TP-315 based on concentration-dependent inhibition of CYP2B6 (**a**), CYP2D6 (**b**), CYP3A4 (**c**), CYP3A5 (**d**), and CYP2C19 (**e**) at 0.015, 0.1, 0.5, 1, 2.5, 5, and 10 μg/mL. Ketoconazole at 10 µM, quinidine at 10 µM, or miconazole at 30 µM were included as positive inhibitors. Data are presented as percent control activity (%). The data were plotted as the mean value ± standard error (SD) and analyzed using GraphPad v.5.01 (* statistically significant one-way ANOVA with *p* < 0.05).

In this study, fluorescence tests were used to determine the effect of TP-315 on the metabolism of drugs mediated by the cytochrome CYP450 enzyme system. A concentration-dependent screening of TP-315 was performed to verify whether TP-315 inhibited the enzymes of CYP2B6, CYP2D6, CYP2C19, CYP3A4, and CYP3A5. TP-315 at a concentration of 0.015 μg/mL (similar to the concentration of the compound measured in the serum of the mice) did not statistically significantly inhibit the activity of the enzymes CYP2B6 (Figure 8a), CYP2D6 (Figure 8b), CYP3A4 (Figure 8c), and CYP3A5 (Figure 8d) compared to the control (*p* > 0.05).TP-315 at a concentration of 0.015 µg/mL statistically significantly inhibited the activity of the CYP2C19 enzyme compared to the control (*p* < 0.05) (Figure 8e).

The CYP2C subfamily accounts for approximately 20% of the CYP450 superfamily of enzymes in the human liver. These enzymes have a protein structure, they are mono-oxygenases that catalyze the synthesis of cholesterol and steroids and the metabolism of drugs [47]. The enzyme CYP2C19 is involved in the metabolism of many groups of drugs: antidepressants (escitalopram), antivirals (nelfinavir), proton pump inhibitors (lansoprazole, omeprazole), cytotoxic agents (teniposide, cyclophosphamide), antiplatelet drugs (clopidogrel), antifungal agents (voriconazole), anxiolytics (diazepam), beta-blockers (propranolol), and anticonvulsants (mephenytoin—used as a probe) [48]. Inhibition of the activity of the enzyme CYP2C19 by TP-315 may lead to the inhibition of the metabolism of the abovementioned drugs and the occurrence of toxic effects. When analyzing the role of TP-315 as a potential anticonvulsant drug, the potential for interaction with drugs metabolized by the enzyme CYP2C19 should be taken into account.

## 3. Materials and Methods

### 3.1. Synthesis of 1,2,4-Triazole-3-Thione Derivatives

Four 1,2,4-triazole-3-thione derivatives, depicted in Figure 1, were synthesized as described in the published articles [10,11,13,15]. In brief, the respective carboxylic acid hydrazides were reacted with aryl/aryl isothiocyanates in order to obtain 1,4-disubstituted thiosemicarbazide derivatives. Their alkaline dehydrocyclization in 2% NaOH produced the respective 4,5-disubstituted-1,2,4-triazole-3-thione derivatives. The obtained compounds were crystallized from EtOH, and their structures were confirmed on the basis of 1H- and 13C-NMR spectra (Bruker Avance, 300 MHz). Reagents and solvents were purchased from Sigma-Aldrich (St. Louis, MO, USA) and POCh Gliwice (Gliwice, Poland), respectively.

### 3.2. Parallel Artificial Membrane Permeability ASSAY (PAMPA BBB)

BBB permeability of the compounds was investigated by using a PAMPA method (parallel artificial membrane permeability assay). The PAMPA system consisted of a 96-well microfilter plate and a 96-well filter plate and was divided into two chambers: a donor at the bottom and an acceptor at the top, separated by a 120-μm-thick microfilter disc coated with BBB lipid solution (Pion, Inc.). The solutions of each compound were prepared in dimethyl sulfoxide (DMSO) at 4 mg/mL concentration and then diluted with Prisma buffer (pH = 7.4) to obtain the donor drug solution with the final nominal concentration of 20 µg/mL. The donor solutions were placed on the donor plate. Acceptor plate contained Brain Sink Buffer (BSB). The plates were put together and incubated at 37 °C for 180 min in a humidity-saturated atmosphere. The concentrations of the compounds were determined with a UV-reader (Multiskan GO, Thermo Scientific) at 254 nm in the donor and acceptor compartments.

The permeability values (Pe) were calculated by using the following equation:

(1) $$\frac{-ln\left(1-\frac{C_{A}}{C_{equilibrium}}\right)}{S\times \left(\frac{1}{V_{D}}+\frac{1}{V_{A}}\right)\times t}$$

where *V_D_*: donor volume, *V_A_*: acceptor volume, *C_equilibrium_*: equilibrium concentration $C_{equilibrium}=\frac{C_{D}\times V_{D}+\;C_{A}\times V_{A}}{V_{D}+V_{A}},\;$CD: donor concentration, *C_A_*: acceptor concentration, *S*: membrane area, and *t*: incubation time (in seconds). High BBB permeation (CNS+) was expected for compounds with Pe > 5.19, whereas low BBB permeation (CNS-) was expected for compounds with Pe < 2.07 [21].

### 3.3. Experiments on Adult Male Albino Swiss Mice

Before starting the experimental part of the project using adult male Albino Swiss mice, the consent of the Local Ethical Committee for Animal Experiments in Lublin (Resolution No. 71/2019) was obtained. The experiments were performed in the certified Center of Experimental Medicine of the Medical University of Lublin. The animals were kept in fixed groups under standard conditions in accordance with the regulation of the Minister of Agriculture and Rural Development as of December 14th, 2016 on minimum requirements to be met by the institution and minimum requirements for the care of animals kept (Journal of Law, item 2139, Poland). The vivarium conditions were as follows: a constant temperature of 20–24 °C, humidity of 45-65%, and a 12-h lighting cycle (6:00–18:00 daylight, 18:00–6:00 night). The experiments started after a minimum of 7 days of acclimatization.

The experiments were carried out on adult male Albino Swiss mice weighing 20 ± 5 g. The mice from the experimental groups (16 mice) were administered with TP-315, which, in the PAMPA BBB assay, showed the highest permeability across the blood–brain barrier. TP-315 was administered intraperitoneally (i.p.) for 14 days at the ED_50_ dose (47.6 mg/kg body mass) determined in the previously conducted preliminary studies [11]. The mice from the control group (8 mice) received 0.9% NaCl solution i.p. once daily for 14 days.

After completion of the experimental procedure (on the 15th day—24h after the last injection), the animals were decapitated. Their livers and kidneys were collected for histopathological examination, while blood was drawn for biochemical and morphological tests and for HPLC-FL determination of TP-315 in serum.

### 3.4. Quantification of TP-315 by HPLC-FL Method

- Stock and working standard solutions

The working standard solutions at a calibration range of 0.05–0.2 mg/L (0.05, 0.08, 0.1, 0.15, 0.18, and 0.2 mg/L) were prepared by appropriate dilution of the 100 mg/L stock solution in methanol (POCh Gliwice, Poland). Working solutions were also prepared for the quality control (QC) samples by the dilution of stock solution to obtain a final concentration of 0.06, 0.12, and 0.18 mg/L. Stock solutions and working solutions were stored in the refrigerator at 4 °C.

- Sample preparation

Serum samples for recovery study and quantification of the examined compound using the standard addition method were prepared by spiking 0.1 mL of TP-315 free serum or the serum of treated mice with 0.4 mL deionized water and 0.5 mL of the appropriate standard solution, providing a calibration range between 0.0125–0.05 mg/L (0.0125, 0.025, 0.0375, and 0.05 mg/L) and QC levels of 0.06, 0.12, and 0.18 mg/L. Investigated serum samples were treated in the same manner as the QCs.

- Sample extraction

Obtained mixture was heated at a temperature of 60 °C for 5 min in an ultrasonic water bath and then centrifuged at 9000× *g* for 15 min. The supernatant of each aliquot of serum was loaded onto the conditioned SPE column. Previous examination has proven that there is no evidence that the conditions applied in the sample preparation led to any degradation of the investigated compound [45]. Solid-phase extraction (SPE) was carried out using BAKERBONDTM speOctadecyl (C18) cartridges (200 mg, 3 mL) on a Baker spe-12G apparatus. The C18 cartridges were activated and conditioned with 2 × 1 mL of methanol and 2 × 1 mL of water. The aliquots of serum were loaded onto the conditioned column. Then, they were washed with 2 × 1 mL of water and dried, applying full vacuum for 1 min. Analyte was eluted with 2 mL of acetonitrile and 20 µL of the eluate was injected directly into the HPLC column.

- HPLC-FL conditions

The HPLC analysis was carried out using an Elite LaChrom HPLC Merck-Hitachi (Merck, Darmstadt, Germany) equipped with a fluorescence detector (L-2485U) and a column thermostat Jetstream 2 Plus (100375, Knauer). Chromatographic separation was carried out at 20 °C using a reversed-phase Zorbax Extend-C18 column (150 mm × 4.6 mm I.D., 5-μm, Agilent Technologies). The mobile phase was methanol (80%, *v/v*) and perchloric acid (0,1%, *v/v*) (Merck, Darmstadt, Germany). The flow rate of the mobile phase was 1 mL/min^−1^. The fluorescence detection was performed at an excitation of 265 nm and an emission of 411 nm. The detection conditions were taken from previous articles [45,46]. The injection volume was 20 µL, corresponding to the volume of the Rheodyne injector loop.

- Validation procedure

The linearity and precision of the HPLC-SPE method were established. Graphs were constructed of the analyte’s peak area against concentration according to ICH requirements [49]. For the construction of the calibration relationship, spiked serum samples at four concentrations were prepared and analyzed in three independent analytical runs. Linear regression analysis was applied to create the equation of the calibration curves. The analyte concentration in the serum sample was determined from the relationship between the added standard of TP-315 concentration and the peak area, extrapolated to the x axis.

### 3.5. Histopathological Examinations of Liver and Kidney Samples Collected from the Examined Mice

The kidneys and livers of mice from the control and experimental groups were subjected to histopathological evaluation. The organs were fixed in 10% buffered formalin at pH 7.2 and then fixed with increasing concentrations of alcohol solutions, acetone, and xylene in paraffin blocks using a tissue processor (Leica TP-20, Leica Biosystems, Wetzlar, Germany). 4 μm thick tissue sections cut with a sledge microtome (Leica SR-200, Leica Biosystems, Wetzlar, Germany) were transferred onto slides. The preparations for the histopathological samples were stained with hematoxylin and eosin and evaluated under a light microscope (Nikon Eclipse E-600, Zeiss, Oberkochen, Germany). Reagents were purchased from Sigma-Aldrich (St. Louis, MO, USA).

### 3.6. Determination of Morphological and Biochemical Parameters

For the determination of biochemical parameters (ALT, AST, GGT, creatinine, urea), whole blood was collected in clotting activator tubes (Sarstadt, Nümbrecht, Germany). To obtain serum, blood tubes were centrifuged at 4200 RPM for 10 min. Biochemical determinations were performed on an ERBA XL 640 analyzer (ERBA Mannheim, Germany) according to the manufacturer’s standard procedure. Whole blood for the determination of morphological parameters was collected in ethylenediaminetetraacetic acid (EDTA) tubes (Sarstadt, Nümbrecht, Germany). Morphological parameters were determined on the MYTHIC 18 Cormay analyzer (Cormay, Lublin, Poland) according to the manufacturer’s standard procedure.

### 3.7. Determination of CYP450 Activity with VIVID CYP450 Assay Kits

Possible inhibitory effect of TP-315 on the catalytic activity of human cytochrome CYP450 enzymes was tested using Vivid CYP450 screening kits (Thermo Fisher Scientific, Waltham, MA, USA) according to the manufacturer’s standard procedure (Vivid BOMCC substrate CYP2B6 blue, Vivid DBOMF substrate CYP3A4 green, Vivid DBOMF substrate CYP3A5 green, Vivid EOMCC substrate CYP2C19 blue, Vivid MOBFC substrate CYP2D6 cyan). The experiment was carried out in the endpoint test mode (reading after 20 min of incubation). The test compound was dissolved in DMSO (starting concentrations, 2.5× concentrated); to avoid the influence of the solvent on the enzyme activity, the results were compared with the solvent control. The influence of TP-315 on the activity of selected enzymes was tested at concentrations of 0.015, 0.1, 0.5, 1, 2.5, 5, and 10 μg/mL. The positive control compounds (inhibitors) were miconazole (for CYP2C19 and CYP2B6), quinidine (for CYP2D6), and ketoconazole (for CYP3A4 and CYP3A5). Inhibitor concentrations were selected according to the manufacturer’s recommendations to achieve ≥90% inhibition. All inhibitors were obtained from Sigma-Aldrich (St. Louis, MO, USA). The BioTek Synergy H1 reader (BioTek Instruments, Inc., USA) was used to read the fluorescence according to the manufacturer’s guidelines. The tests were performed in duplicate.

### 3.8. Methods of Statistical Data Analysis

Statistica v.13.3 and GraphPad v.5.01 were used in the statistical analysis and graphic design (*p* < 0.05 was assumed statistically significant). Unpaired t-test was used to calculate the differences for the values of biochemical and morphological parameters between the experimental group and the control group. One-way ANOVA test was used to calculate statistical significance in the enzyme assay.

## 4. Conclusions

TP-315 has a high permeability across the blood–brain barrier in in vitro tests (approximately 185 times greater in comparison to valproic acid). On the basis of both histopathological and biochemical examination, it was found that TP-315 exhibits neither hepatotoxic nor nephrotoxic effects after long-term use in mice. Based on the morphological examination, it was found that TP-315 did not cause changes in the white cell and red cell systems of mice chronically treated with the test compound. In vitro tests showed that TP-315 did not inhibit CYP2B6, CYP2D6, CYP3A4, or CYP3A5 enzymes at the concentration found in the serum of mice (≈0.015 µg/mL) subjected to long-term exposure to this compound.

## Figures and Tables

**Table 1:** In vitro measurement of the ability of the investigated 1,2,4-triazole derivatives to permeate through the BBB.

Compound	Permeability Coefficients (Pe × 10−6) ± SD [cm/s]
Valproic acid	15.37 ± 4.71
TP-10	40.2 ± 5.1
TP-315	2983.50 ± 211.02
TP-427	476.63 ± 39.59
TPR-22	34.43 ± 8.30

**Table 2:** The comparison of neurotoxicity (chimney test and rotarod test), affinity towards batrachotoxin-binding site on sodium channels, and anticonvulsant activity (MES test and 6 Hz test) of selected 1,2,4-triazole-3-thionie derivatives based on previously published studies [10,11,12,13,18,22].

Compound	Pretreatment Time (min)	Anticonvulsant Activity in MES Test [10,11,13]		Anticonvulsant Activity in 6 Hz Test [12]		Neurotoxicity in Chimney or Rotarod (†) Tests in Mice [10,11,13]	Affinity towards Batrachotoxin-Binding Site on Sodium Channels [11,18,22]
		ED_50_ ± S.E. [mg/kg]	PI (TD_50_/ED_50_)	ED_50_ ± SEM [mg/kg]	PI (TD_50_/ED_50_)		IC50 (μM) ± SEM
TP-10	15	57.0 ± 9.4	5.9	62.6 ± 13.2	5.4	338.1 ± 12.0	no data
	30	74.5 ± 8.1	4.5	61.1 ± 9.7	5.5	338.1 ± 14.7	
	60	187.1 ± 18.8	1.8	169.7 ± 18.5	2.0	333.4 ± 18.6	
	120	281.4 ± 13.6	1.4	167.6 ± 17.4	2.4	395.1 ± 25.2	
TP-315	15	47.6 ± 3.8	9.7	61.3 ± 10.1	7.6	462.9 ± 20.0	6.21 ± 0.80
	30	68.3 ± 10.3	6.8	59.7 ± 6.8	7.8	462.9 ± 20.0	
	60	98.1 ± 16.4	4.7	68.1 ± 11.0	6.7	456.9 ± 19.7	
	120	159.7 ± 21.7	2.8	136.2 ± 18.3	3.3	448.1 ± 21.7	
TP-427	15	72.1 ± 7.0	>13	40.9 ± 6.4	>24.4	>1000	6.17 ± 1.43
	30	74.5 ± 8.1	>13	46.6 ± 8.2	>21.5	>1000	
	60	83.6 ± 3.8	6.5	51.6 ± 6.9	10.5	540.7 ± 20.9	
	120	97.9 ± 10.9	5.6	64.9 ± 5.6	8.5	548.5 ± 21.4	
TPR-22	15	130.4 ±17.6	2.3	no data	no data	306.0 ± 19.8 (†)	18.9 ± 1.2
	30	130.4 ± 17.6	2.4	no data	no data	314.5 ± 22.0 (†)	
	60	159.9 ± 21.9	2.0	no data	no data	325.9 ± 23.1 (†)	
	120	195.7 ± 21.5	1.7	no data	no data	329.9 ± 24.2 (†)	

**Table 3:** The level of peripheral blood morphological parameters in mice from the control and experimental groups.

Parameter	Unit	Control Group	Examined Group	*p* Value
RBC	10*6/μL	8.24 ± 0.44	8.37 ± 0.84	0.8183
HGB	g/dL	14.03 ± 0.35	13.88 ± 1.28	0.8499
MCV	µm3	49.53 ± 2.04	49.1 ± 20.06	0.0504
MCH	pg	17.0 ± 0.72	16.62 ± 0.38	0.3536
MCHC)	g/dL	34.33 ± 0.06	33.84 ± 0.46	0.1244
RDW-CV	%	13.27 ± 1.49	13.46 ± 0.56	0.9881
RDW-SD	µm3	28.3 ± 2.61	28.46 ± 1.36	0.4434
HCT	%	40.77 ± 0.95	41.08 ± 3.65	0.6810
PLT	10*3/µL	879.0 ± 64.21	1034.8 ± 268.44	0.3285
MPV	µm3	5.2 ± 0.2	5.08 ± 0.25	0.3336
PDW		15.5 ± 0.36	15.24 ± 0.17	0.0740
PCT	%	0.35 ± 0.17	0.53 ± 0.16	0.1874
WBC	10*3/µL	4.46 ± 1.01	4.25 ± 1.2	0.8096
BAS	%	0.43 ± 0.22	0.44 ± 0.19	0.5824
NEU	%	22.53 ± 2.4	23.28 ± 7.19	0.8709
LYM	%	69.0 ± 2.61	65.7 ± 6.96	0.4714
MON	%	3.93 ± 0.48	4.04 ± 1.17	0.8881
EOS	%	4.1 ± 0.53	6.54 ± 2.05	0.0972

**Table 4:** The recoveries of TP-315 from the blank serum samples with the RSD% values of intra- and inter-day precision.

Analyte Concentration in the Injected Sample [ppm]	Extraction Yield [%±SD]	Repeatability [RSD%]	LOD [ppm]	LOQ [ppm]
0.06	96.66 ± 1.77	1.83	0.000025	0.000084
0.12	99.39 ± 0.44	0.44		
0.18	97.46 ± 0.94	0.97		

## Data Availability

The data that support the findings of this study are available from the corresponding author upon reasonable request.
